# Long noncoding RNA Linc00337 functions as an E2F1 co-activator and promotes cell proliferation in pancreatic ductal adenocarcinoma

**DOI:** 10.1186/s13046-020-01725-5

**Published:** 2020-10-14

**Authors:** Huakai Wang, Shiyong Yu, Huan Peng, Yijun Shu, Wenjie Zhang, Qi Zhu, Yingxia Wu, Yijun Xu, Jiqi Yan, Honggang Xiang

**Affiliations:** 1 Department of General Surgery Pudong New Area People’s Hospital Pudong New Area, No. 490, South Chuanhuan Road, Shanghai, 201200 China; 2 Department of General Surgery, Xinhua Hospital affiliated to Shanghai Jiaotong University School of Medicine, No. 1655, Kongjiang Road, Shanghai, 200092 China; 3 Department of General Surgery, Ruijin Hospital, Shanghai Jiao Tong University School of Medicine, Shanghai, 200025 China

**Keywords:** PDAC, Long noncoding RNA, Linc00337, E2F1, Cell cycle, Cell proliferation

## Abstract

**Background:**

Long noncoding RNA (lncRNA) Linc00337 has been implicated in lung, gastric, colorectal and esophageal squamous cell carcinoma progression via various mechanisms; however, its clinicopathological significance and role in pancreatic ductal adenocarcinoma (PDAC) progression remains largely unknown.

**Methods:**

Multiple approaches such as bioinformatic analysis, Transfection, quantitative real-time-PCR, Western blotting, animal studies, RNA-immunoprecipitation (RIP), RNA-pulldown and RNA-Fluorescence in situ hybridization (RNA-FISH) and were utilized to explore the role of Linc00337 in PDAC.

**Results:**

Here we identified Linc00337 is an oncogenic lncRNA during PDAC progression. We found that the expression of Linc00337 is elevated in PDAC tissues and the higher Linc00337 predicts dismal prognosis. Functionally, Linc00337 promotes PDAC cell proliferation and cell cycle transition both in vitro and in vivo. Mechanistically, Linc00337 binds to E2F1 and functions as an E2F1 coactivator to trigger the targets expression during PDAC progression.

**Conclusion:**

Our results demonstrate a reciprocal regulation mechanism between Linc00337 and E2F1 in PDAC progression and report the clinical value of Linc00337 for PDAC prognosis and treatment.

## Background

Pancreatic ductal adenocarcinoma (PDAC) accounts for approximately 90% of all pancreatic carcinomas [1] and has a poor five-year survival rate of 2–9% [2] owing to a lack of early symptoms and early detection and most cases being diagnosed at an advanced stage. PDAC tumorigenesis and progression are the result of a series of gene mutations and previous studies have revealed that KRAS, P16, CDNK27, P53, and SMAD4 mutations contribute toward PDAC development [3, 4]; however, the detailed molecular pathogenesis of PDAC remains largely unknown.

Cell cycle deregulation is a common feature of human cancer [5], with fundamental mutations in the genetic control of cell division resulting in spontaneous proliferation [6]. For instance, P16 and P53 mutations release the inhibition of CDK4/CDK6-mediated RB phosphorylation and trigger E2F activation-mediated cell cycle progression [7, 8]. Aberrant high E2F expression has been reported in many malignant cancers, including gastric, colorectal, breast, and lung cancers, and E2F dysregulation has been shown to contribute toward unrestrained cell proliferation and cancer development [9].

Long non coding RNAs (lncRNAs), which function primarily via interaction partners such as chromatin DNA, proteins, and RNAs [10, 11] have recently attracted widespread attention as a new coregulator of cancer development [12]. Numerous oncogenic and tumor-suppressive lncRNAs have been reported to regulate tumor cell proliferation, growth, metabolism, and metastasis, thus are regarded as potential biomarkers and therapeutic targets for cancer diagnosis and treatment [13, 14]. Although some E2F1-responsive lncRNAs have been shown to play key roles in cell proliferation and cell cycle transition [15–18], none have directly regulated E2F1 transactivation.

The lncRNA Linc00337 (accession number: NR_103534.1) has been shown to promote gastric cancer, lung cancer, colorectal cancer and esophageal squamous cell carcinoma progression cell proliferation [19–22]; however, it has been poorly studied and its roles and underlying mechanisms in PDAC remain largely unclear. In this study, we conducted a comprehensive survey of cancer related lncRNAs in The Cancer Genome Atlas (TCGA) PDAC database and found that Linc00337 is significantly upregulated in PDAC and predicts poor prognosis. Therefore, we investigated the interaction between Linc00337 and E2F1 and the role of Linc00337 in PDAC by detecting Linc00337 expression in paired PDAC tissues and PDAC cells. Thus, our study suggests that Linc00337 could be used as a potential prognostic biomarker and therapeutic target for PDAC.

## Materials and methods

### Clinical specimens

Fresh paired PDAC tissues were collected from 18 cases of PDAC surgical resection at the Department of General Surgery, Xinhua Hospital, with informed consent. Normal tissues were defined as that 2 cm away from malignant tissues. The patient inclusion criteria were as follows: (1) aged 46–70; (2) no other types of tumor or diseases; (3) had not undergone preoperative chemotherapy or radiation therapy. All tissues were obtained under sterile conditions during surgery, flash frozen in liquid nitrogen, and stored at − 80 °C.

### Cell lines

The normal pancreatic ductal cell line HPDE and PDAC cell lines AsPC1, BxPC3, MiaPaCa2, and PANC1 were purchased from the American Type Culture Collection (ATCC; Manassas, VA, USA). All cell lines were maintained in DMEM with high glucose and sodium pyruvate, except for AsPC1 which was maintained in 1640. All media were supplemented with 10% fetal bovine serum (Sigma), 100 units/mL of penicillin and 100 μg/mL of streptomycin (Gibco).

### Lentiviral infection and establishment of stable cell lines

Linc00337 (pLVX-337, pLVX-E2F1) or empty (pLVX) vectors were purchased from GeneCopoeia (Guangzhou, China), and Linc00337-targeting (sh#1, sh#2, sh#3, shE2F1) or negative control (NC) shRNAs were obtained from RiboBio (Guangzhoug, China). The lentivirus was constructed in HEK-293 T cells and collected from the supernatant after 24 and 48 h. AsPC1 or PANC1 cells were infected with the lentiviruses and selected with 2 μg/mL of puromycin (Millipore, USA) 48 h after infection.

### RNA isolation, RNA-seq, quantitative real-time PCR (qRT-PCR)

Total RNA was isolated from tissues and cells using TRIzol reagent (Invitrogen, USA) according to the manufacturer’s instructions and subjected to RNA-seq analysis by Shanghai Kangcheng Biotech. Raw data were deposited in the GEO database (accession number GSE146671). For qRT-PCR, 2 μg of total RNA was treated with DNase I and reverse transcribed using an MMLV system (Promega, USA). qRT-PCR was performed using an ABI 7900 RT-PCR system with SYBR Green Real-time PCR Master Mix (ABI, USA) and 18S RNA as a control. Primer sequences are listed in Table 1.

**Table 1:** primers and shRNA target sequences

# qRT-PCR primers (5′-3′)		
	Forward	Reverse
337 V1	CTCTGATCTGTCCACCCTCG	TTCCTGGGGTTTGTGTTCGG
337 V2	GCGAATCATTTGAGTAGAGA	CAAGTGAAGTCAGGATCACA
337 V1 + V3	AGTTGCTGGAGTTGCCGAAT	TCTCCGTGTGTGTGTTTCCC
18S	GTAACCCGTTGAACCCCATT	CCATCCAATCGGTAGTAGCG
GAPDH	GGAGCGAGATCCCTCCAAAAT	GGCTGTTGTCATACTTCTCATGG
JUNB	ACAAACTCCTGAAACCGAGCC	CGAGCCCTGACCAGAAAAGTA
JUND	TCATCATCCAGTCCAACGGG	TTCTGCTTGTGTAAATCCTCCAG
CCNB1	AATAAGGCGAAGATCAACATGGC	TTTGTTACCAATGTCCCCAAGAG
CCNB2	CCGACGGTGTCCAGTGATTT	TGTTGTTTTGGTGGGTTGAACT
MYC	GGCTCCTGGCAAAAGGTCA	CTGCGTAGTTGTGCTGATGT
ChIRP used primers (5′-3′)		
CCNB1	TGTGACCCTGGCAAAGTCAT	CAAGAGTGTGCGTTGCCAAT
JUNB	ATGCGTACCCCGAGGTCCTTTGA	AGCCTGAGCCACACGCCTTTATA
MYC	TTTATAATGCGAGGGTCTGG	TATTCGCTCCGGATCTCCCTT
FOXM1	GAGCTTTGAAAAGGGGAGCA	ACCGGAGCTTTCAGTTTGTT
shRNA target sequenses (5′-3′)		
337 sh#1	CCTCCCAAAGTGCTGAGATTA	
337 sh#2	CCTCCCAAAGTGCTGAGATTA	
337 sh#3	GTACCACTTTATTTTATTTTA	
E2F1 shRNA	AGCTGGACCACCTGATGAATA	
	ChIP-qRT-PCR primers	
	Forward	Reverse
337BS1	CCAAGTAGCTAGAATTACAGC	TTTAATCCCAGCACTTTGGGA
337BS2	TGCGCACTGTGCCGCCGATGC	CTCCTGAGTTTGGGACTAAGT
U1	ATACTTACCTGGCAGGGGA	AGGGGAAAGCGCGAACGCAG
MYC	AGATCCTCTCTCGCTAATCT	ATACTCAGCGCGATCCCTCC

### In vitro transcription/translation assays

In vitro translation assay was performed using the TnT Quick Coupled Transcription/Translation System (Promega), according to the manufacturer’s instructions. Reactions were carried out using, 1 mM transcend biotin-lysyl-tRNA. The translation products were then separated on 10% SDS-PAGE gels, transferred onto nitrocellulose membrane and visualized by binding of streptavidin–horseradish peroxidase, followed by chemiluminescent detection. The assay was carried out on by pcDNA3.1(+) vector containing the full length of Linc00337 (V2), HOTAIR and the CDS sequences of MYC. HOTAIR functions a negative control, MYC as positive control.

### Western blot

Cells were seeded into 6 cm plates (4 × 10^5^ cells/plate) for 48 h and the lysates were subjected to SDS-PAGE electrophoresis, transferred onto a nitrocellulose membrane, and blocked with 5% skim milk at room temperature. The membranes were incubated with primary antibodies overnight, washed with phosphate buffered saline (PBS), and incubated with anti-mouse (Abcam, ab6728) or rabbit IgG-HRP (Abcam, ab6721). The primary antibodies were as follows: E2F1 (Abcam, ab218527), AURKB (Abcam, ab24), DSN1 (Proteintech, 17,742–1-AP), CCNB2 (Abcam, ab185622), CHEK1 (Abcam, ab40866), CENPA (Abcam, ab13939), DP1 (Abcam, ab124678), β-actin (Santa Cruz Biotechnology, sc69879), and GAPDH (Proteintech, 10,494–1-AP).

### CCK8 cell viability assays

Approximately 2000 cells were seeded into 96-well plates and their absorption was measured every 24 h at 450 nm using a CCK-8 kit (Dojindo Laboratories, Kumamoto, Japan) according to the manufacturer’s instructions.

### Colony formation assays

Cells (500 cells/3 mL) were seeded into 12-well plates and incubated for 8–12 days at 37 °C with 5% CO_2_, with the medium changed every other day. Colonies were washed once with PBS, fixed with 0.5% paraformaldehyde for 20 min, and stained with crystal violet before the number of clones was counted. All assays were conducted in triplicate and results were expressed as the mean value.

### Cell cycle analysis

The indicated cells were washed with 1× PBS, trypsinized, and collected before being fixed with 70% pre-coated ethanol at − 20 °C until further examination. Prior to analysis, samples were treated with 20 mg/mL of RNase (Sigma-Aldrich) for 1 h at 37 °C, labeled with 20 mg/mL propidium iodide (Sigma-Aldrich), and assessed by FACS Calibur flow cytometry (BD).

### RNA-FISH assays

To detect the subcellular distribution of Linc00337, RNA-FISH assays were conducted. Briefly, cells were fixed with 4% PFA for 15 min, permeabilized with 0.5% Triton X-100 for 5 min on ice, and then incubated with RNA-FISH probes (RiboBio) in hybridization buffer at 37 °C overnight. Nuclei were counterstained with DAPI.

### Dual luciferase reporter assays

To determine the effect of E2F1 on the Linc00337 promoter, stable E2F1-knockdown PANC1 cells and AsPC0-overexpressing cells were transfected with the PGL3- 337BS1/337BS2 construct and a Renilla luciferase reporter plasmid. After 24 h, firefly and Renilla luciferase activity were measured using a Dual Luciferase Reporter Assay System (Promega). To evaluate the effect of Linc00337 on E2F1 transcriptional activity, the cells were co-transfected with an E2F or E2F (mut) luciferase reporter plasmid (YEASEN) and Renilla plasmid. After 24 h, firefly and Renilla luciferase activity were measured using the Dual Luciferase Reporter Assay System (Promega).

### RNA pull-down assay

Biotin-labeled RNA pull-down assays were performed as described previously. Briefly, Linc00337 and linc00337-AS were transcribed in vitro from linearized constructs using a Biotin-RNA Transcription Kit (Roche) and purified with TRIzol reagent (Invitrogen) before being incubated in RNA structure buffer (10 mM Tris-HCl pH 7, 0.1 M KCl, 10 mM MgCl_2_) and heated to 72 °C for 2 min to form proper secondary structures. The RNAs were then incubated with PANC1 cell lysates at 4 °C for 4 h followed by streptavidin beads (Thermo) at room temperature for 1 h, washed five times, and analyzed by western blot.

### RNA immunoprecipitation assay (RIP)

RIPA was performed as described previously. Briefly, 2 × 10^7^ PANC1 cells were crosslinked with 0.3% formaldehyde in medium for 10 min at room temperature, neutralized with 0 mM glycine for 5 min, and washed twice with cold PBS. Next, the cells were lysed in RIPA buffer (50 mM Tris pH 7.4, 150 mM NaCl, 1 mM EDTA, 0.1% SDS, 1% NP-40, 0.5% sodium deoxycholate, 0.5 mM dithiothreitol, RNase and protease inhibitor cocktail), sonicated on ice, and treated with DNase before being pre-cleared with protein A/G beads (Thermo) for 30 min and incubated with Flag antibodies (Sigma, F3165) or mouse IgG (Abcam, ab190475) at 4 °C overnight. Antibodies were precipitated by incubation with protein A/G beads, washed five times for 10 min, and then RNA was extracted with Trizol reagent (Invitrogen) and detected by qRT–PCR. Protein samples were then subjected to western blotting.

### Subcutaneous tumor formation assays

Stably-transfected cells (5 × 10^6^ cells) were subcutaneously injected into both flanks of at least five 6-week-old female BALB/c nude mice (Linchang Biotech). After 24 days, the mice were sacrificed and imaged. Animal care and experiments were performed in strict accordance with the “Guide for the Care and Use of Laboratory Animals” and were approved by the Committee for the Humane Treatment of Animals at Shanghai University of Medicine & Health Sciences. And the volume of tumor was calculated by the formula: L × W^2^ (L: the longest diameter of the tumour, W: the shortest diameter of the tumour).

### Immunohistochemical (IHC) staining

Tumor slides were stained according to standard IHC protocols. Briefly, slides were blocked with 10% bovine serum albumin (Sangon) for 1 h, incubated with PCNA antibodies (Abcam, ab18197) overnight at 4 °C, and then incubated with HRP-labeled secondary antibodies (Dako) for 1 h at 25 °C. Antibodies were detected using diaminobenzidine substrate chromogen (DAB). All slides were counterstained with hematoxylin before dehydration and mounting.

### Chromatin immunoprecipitation (ChIP) assays

ChIP assays were performed using a Pierce Agarose ChIP Kit according to the manufacturer’s protocol. PANC1 cells were crosslinked with 1% formaldehyde for 10 min at 37 °C and then incubated with anti-E2F1 antibodies (Abcam, ab4070) and anti-Rabbit IgG (Abcam, ab2410). Bound DNA fragments were subjected to RT-PCR using specific primers (Table 2).

**Table 2:** Correlations between Linc00337 and key clinicopathological parameters

Variable		Linc00337 (*n* = 18)		
		unchanged	Up-regulated	*Chi-square P* value
Age	≤50 years	1	8	0.527
	> 50 years	2	7	
Gender	Female2	0	3	0.396
	Male1	3	12	
Grade	I	2	0	0.003*
	II and III	1	15	
Tumor size	≤ 2 cm	2	2	0.043*
	> 2 cm	1	13	
TNM stage	T_1_	1	0	0.05*
	T_2_	2	10	
	T_3_-T_4_	0	5	

*p ≤ 0.05

### Chromatin isolation by RNA purification (ChIRP)

ChIRP was performed using PANC1 stable cell lines by adapting previously described protocols [23] with minor modifications. Briefly, PANC1 cells were crosslinked with 1% glutaraldehyde for 10 min at room temperature with gentle shaking. Crosslinking was stopped with 0.25 mM glycine for 5 min. Cross-linked chromatin was incubated with biotinylated 20-mer antisense DNA probes targeting Linc00337 and negative control lacZ RNA (RiboBio) before streptavidin magnetic bead capture and wash/elution steps were performed as described previously [23]. Eluted chromatin and RNA fragments were analyzed by qRT-PCR using the primers listed in Table 1.

### Statistical analysis

Results were expressed as the mean ± standard deviation (SD) of at least three independent experiments. Between-group comparisons were analyzed by Student’s *t*-tests or analysis of variance. *P* values of < 0.05 were considered statistically significant.

## Results

### Linc00337 is highly expressed in PDAC and acts as a prognostic biomarker

To identify oncogenic lncRNAs involved in PDAC progression, we investigated the expression of 20 previously reported cancer-associated lncRNAs (Fig. S1) in PDAC using the online TCGA analysis tool (http://gepia.cancer-pku.cn/). We found that H19, HOXA-AS2, Linc00511, UCA1, Linc00337, CASC15, and CRNDE expression were higher in malignant PDAC tissues than in normal tissues, whereas NEAT1 and MEG3 expression were lower. By analyzing the prognostic significance of these lncRNAs, we found that increased HOTAIR, HOTTIP, and Linc00317 expression predicted poor prognosis, while increased DANCR expression predicted better prognosis (Fig. S2). Several previously reported lncRNAs, such as H19 [24, 25] and HOXA-AS2 [26, 27], were also identified by this analysis; however, we focused on Linc00337 due to its high expression and poor prognostic significance.

**Fig. 1:**
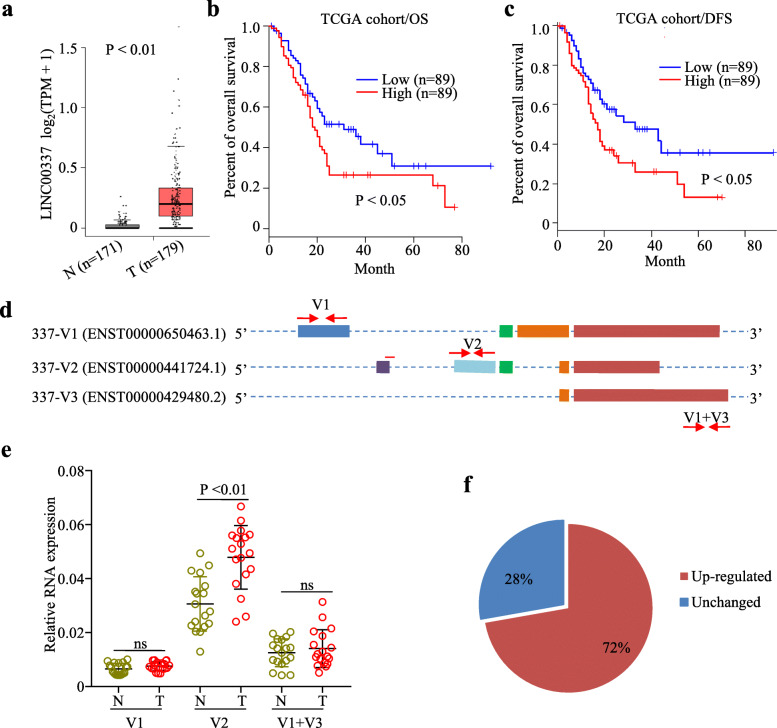
Linc00337 is highly expressed and predicts poor prognosis in PDAC patients. **a**, Linc00337 expression in PDAC (*n* = 179) and normal tissues (*n* = 171). **b-c**, TCGA data depicting that high Linc00337 expression predicts poor overall survival (OS, b) and disease-free survival (DFS, c). **d**, Schematic diagram of Linc00337 variants (337 V1, 337 V2, 337 V3) from the Ensembl database. Red arrows indicate primer locations. **e**, qRT-PCR indicating that 337 V2 is significantly elevated in 18 paired malignant PDAC tissues. **f**, Pie chart depicting Linc00337 V2 distribution in PDAC tissues. N, normal; T, tumor

Compared to normal tissues, Linc00337 was expressed highly in PDAC tissues (Fig. 1a) and predicted poor overall survival (OS; Fig. 1b) and disease-free survival (DFS; Fig. 1c), indicating that Linc00337 could be an oncogenic lncRNA in PDAC tumorigenesis or progression. According to Ensembl database annotation, Linc00337 encodes three variants (337 V1, 337 V2, 337 V3; Fig. 1d); therefore, we investigated the expression of each transcript variant in 18 paired PDAC tissues using specific primers targeting 337 V1, 337 V2, and 337 V1 + V3. As shown in Fig. 1e, qRT-PCR revealed that 337 V2 expression was significantly elevated in PDAC tissues and the 337 V2 expression was remarkably higher than that of 337 V1 and 337 V3. Accordingly, we focused on 337 V2 in subsequent experiments and Linc00337 refers to 337 V2 hereafter.

Next, we analyzed Linc00337 expression in fresh PDAC tissues and found that it was up-regulated in most (72%) malignant tissues, as confirmed by bioinformatic analysis. In addition, clinicopathological analysis revealed that Linc00337 up-regulation correlated significantly with American Joint Committee on Cancer (AJCC) tumor grade, tumor size, and TNM stage (Table 2). Taken together, these findings suggest that Linc00337 might be an oncogenic lncRNA in PDAC.

### Knockdown of Linc00337 inhibits PDAC cell proliferation and tumor growth

**Fig. 2:**
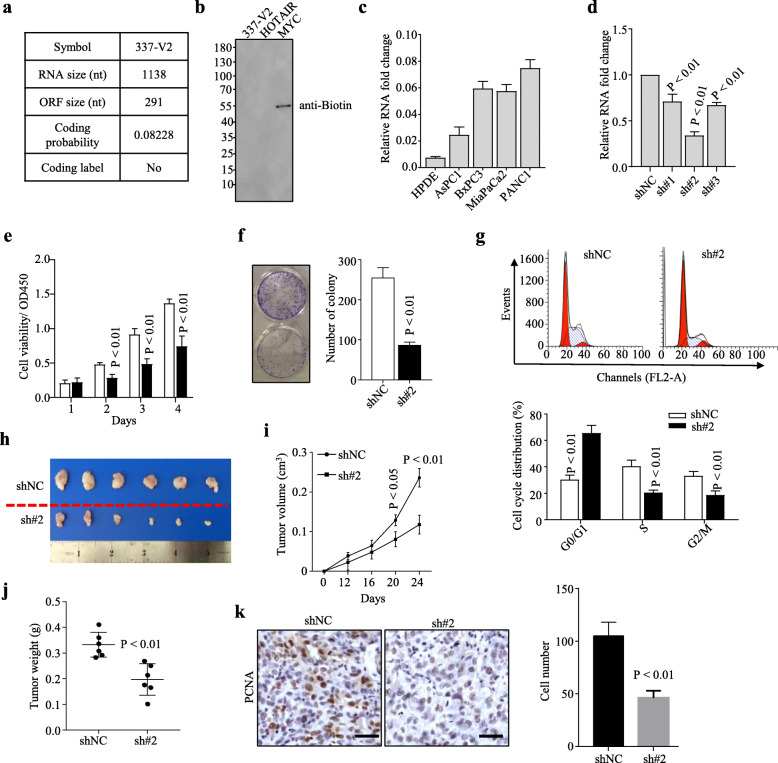
Knockdown of Linc00337 inhibits PANC1 cell proliferation and growth both in vitro and in vivo. **a**, CPAT analysis of Linc00337. **b**, in vitro translation analysis of Linc00337. HOTAIR used as negative control, MYC as positive control. **c**, qRT-PCR analysis of the expression of Linc00337 in indicated cell lines. **d**, qRT-PCR analysis of the expression of Linc00337 in PANC1 cells infected with shNC and shRNAs. **e-f**, knockdown of Linc00337 dramatically inhibits PANC1 cell proliferation and growth as shown by CCK8 cell viability (**e**) and colony formation (**f**) assays. g, depletion of Linc00337 inhibits cell cycle transition from G0/G1 stage to S stage. **h-j**, Xenograft subcutaneous tumor formation assays with stably-transfected PANC1 cells showing tumor size (**h**), growth curve (**i**), weight (**j**), and PCNA staining (**k**). Scale bar, 100 μm. In c-g, data represent the mean ± SD of three independent experiments

To confirm the coding potential of Linc00337, both Coding Potential Assessing Tool (CPAT) prediction (Fig. 2a) and in vitro translation assay (Fig. 2b) were performed, all of these results suggested that Linc00337 is a non-coding RNA.

To investigate the biological roles of Linc00337 in PDAC, we determined its expression in the PDAC cell lines AsPC1, BxPC3, MiaPaCa1, PANC1 and the normal pancreatic ductal cell line HPDE. Linc00337 expression was often elevated in the malignant cell lines (Fig. 2c); therefore, we analyzed the biological effects of Linc00337 in PANC1 tumorigenesis by silencing its expression with three specific shRNAs (sh#1, sh#2, sh#3) in PANC1 cell line and found that sh#2 was much more effective than sh#2 or sh#3 (Fig. 2d). Both CCK8 cell viability assay (Fig. 2e) and cellular colony formation assay (Fig. 2f) revealed that knockdown of Linc00337 impairs cell proliferation and growth. Furthermore, cell cycle assay suggested that depletion of Linc00337 arrests cell cycle in G0/G1 stage (Fig. 2g).

To verify these findings, we further performed subcutaneous tumor formation assays by PANC1 stably transfected cell lines. As illustrated in Fig. 2h-k, the tumor size, tumor weight and PCNA expression were significantly impaired by Linc00337 depletion. All of these findings indicated that Knockdown of Linc00337 inhibits PDAC cell proliferation and tumor growth both in vitro and in vivo.

### Overexpression of Linc00337 promotes cell cycle transition and tumor growth in PDAC

**Fig. 3:**
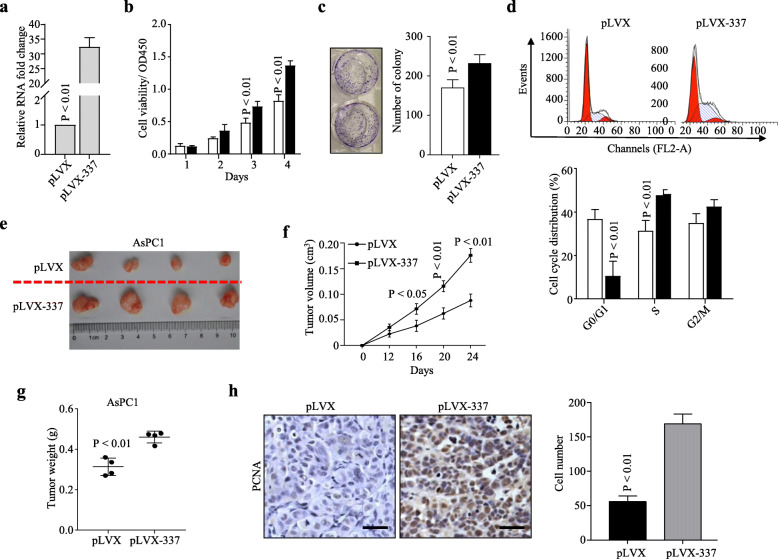
Overexpression of Linc00337 promotes cell cycle transition and tumor growth in PDAC. **a**, qRT-PCR analysis of Linc00337 expression in AsPC1 cells infected with pLVX and pLVX-337. **b** and **c**, Linc00337 overexpression remarkably promotes AsPC1 cell proliferation and growth as shown by CCK8 cell viability (**b**) and colony formation (**c**) assays. **d**, Overexpression of Linc00337promotes cell cycle transition from G0/G1 stage to S stage in AsPC1 cells. **e-g**, Xenograft subcutaneous tumor formation assays with stably-transfected AsPC1 cells showing tumor size (**e**), growth curve (**f**), weight (**g**), and PCNA staining (**h**). Scale bar, 100 μm. In a-d, data represent the mean ± SD of three independent experiments

We also overexpressed Linc00337 in AsPC1 (Fig. 3a). Accordingly, both CCK8 cell viability assay (Fig. 3b) and cellular colony formation assay (Fig. 3c) suggested that overexpression of Linc00337 promotes AsPC1 cell proliferation and growth. Cell cycle assays further confirmed that Linc00337 promotes cell cycle transition (Fig. 3d). Subcutaneous tumor formation assays (Fig. 3e-h) further confirmed that Linc00337 promotes tumor growth in vivo. Collectively, these in vitro assays suggest that Linc00337 significantly promotes PDAC cell proliferation and cell cycle transition.

### Linc00337 regulates cell cycle associated pathways

**Fig. 4:**
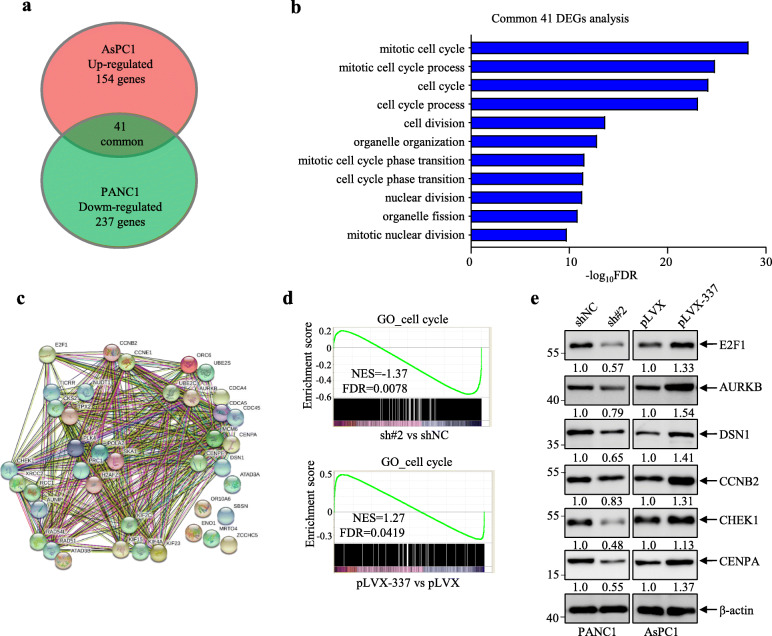
Linc00337 regulates cell cycle associated pathways. **a**, Venn diagram depicting differentially expressed genes (DEGs) regulated by Linc00337 in stably-transfected AsPC1 and PANC1 cells (DEGs with |fold change| > 2, FDR < 0.05). **b** and **c**, GO enrichment (**b**) and string (**c**) analysis of common DEGs showing the top pathways associated with cell cycle and cell division. **d**, GSEA of RNA-seq data from PANC1 (top) and AsPC1 (bottom) stably transfected cells with the indicated gene set. **e**, Western blot analysis of the indicated cell cycle-associated proteins in PANC1 and AsPC1 stable cell lines, the bands were quantified by image J software. In (**a-c**), RNA-seq was repeated twice; in (**d**), experiment was repeated three times

To investigate the role of Linc00337 in PDAC cells, we performed RNA-seq analysis in stably-transfected cell lines (GSE146671). A total of 278 differentially expressed genes (DEGs) were down-regulated by Linc00337 knockdown and 195 were up-regulated by Linc00337 overexpression, with 41 common DEGs in both cell lines (Fig. 4a). Gene ontology (Fig. 4b) and string (Fig. 4c) analysis revealed that the top 11 pathways were associated with the cell cycle and cell division, suggesting that Linc00337 plays a key role in PDAC cell cycle progression.

To validate the RNA-seq analysis, we firstly performed GSEA analysis, as illustrated in Fig. 4d, the altered genes could be enriched in cell cycle associated geneset. we further detected the alterations of cell cycle-related proteins in the stably-transfected PANC1 and AsPC1 cells. Linc00337 knockdown significantly decreased the expression of these proteins in PANC1 cells while Linc00337 overexpression dramatically increased their expression in AsPC1 cells (Fig. 4e). Notably, the important cell proliferation regulator E2F1 was also altered by Linc00337, with RNA decay assays in stably-transfected PANC1 and AsPC1 cells revealing that Linc00337 did not regulate E2F1 mRNA degradation (Fig. S3) and suggesting that Linc00337 regulates E2F1 transcription in PDAC cells. Taken together, these RNA-seq data strongly suggested that Linc00337 promotes PDAC cell proliferation and growth.

### Linc00337 is a direct target gene of E2F1

**Fig. 5:**
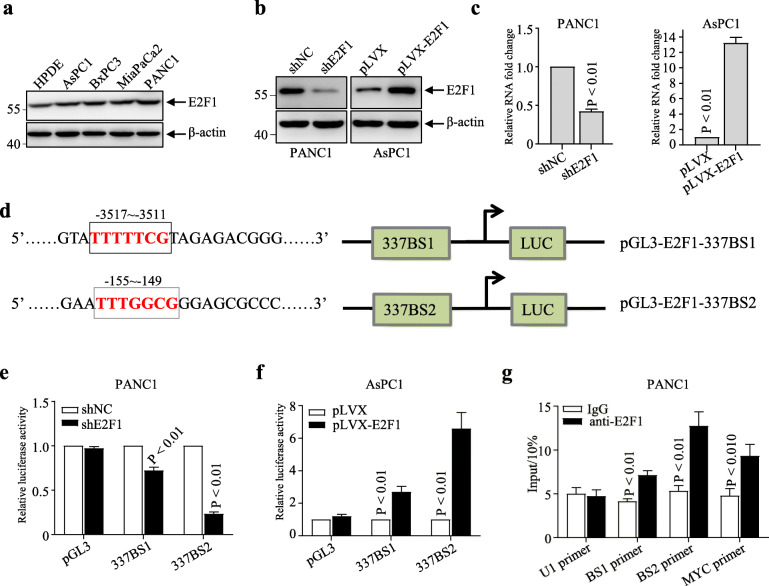
Linc00337 is a direct target of E2F1. **a**, western blot analysis of E2F1 protein levels in the indicated cell lines. **b**, western blot analysis of E2F1 protein levels in the indicated stably-transfected cell lines. **c**, qRT-PCR analysis of Linc00337 in stably-transfected PANC1 (left) and AsPC1 (right) cells. **d**, schematic diagram of E2F1 binding sites in the Linc00337 gene promoter. e and f, barplots depicting the regulation of Linc00337 transcription by E2F1 in PANC1 (**e**) and AsPC1(**f**) cells. **g**, ChIP-qPCR analysis of E2F1 occupancy at the Linc00337 promoter in PANC1 cells. In (**a-c** and **e-f**), data represent the mean ± SD of three independent experiments

Next, we investigated why Linc00337 is up-regulated in PDAC tissues. Since Linc00337 is located in the 1p36.31 genomic region, which is not amplified in PDAC, we investigated potential transcription factors that may target Linc00337 using the JASPAR online database. E2F1 was identified as a potential transcription factor; therefore, we determined its protein levels in HPDE and PDAC cells and found that E2F1 levels were higher in these malignant cell lines (Fig. 5a). Subsequently, E2F1 was silenced in PANC1 cells and overexpressed in AsPC1 cells (Fig. 5b), revealing that E2F1 overexpression and knockdown significantly increased and inhibited Linc00337 expression, respectively (Fig. 5c).

To further illustrate the correlation between E2F1 and Linc00337, we constructed luciferase reporter vectors containing putative E2F1 binding sites (337BS1, 337BS2) in the Linc00337 promoter (Fig. 5d). Dual luciferase reporter assays revealed that luciferase activity was inhibited in PANC1 cells by E2F1 knockdown but elevated by E2F1 overexpression in AsPC1 cells (Fig. 5e and f). ChIP assays were performed to verify these results, suggesting that E2F1 could significantly enrich the 337BS1 and 337BS2 chromatin fragments and the positive control (MYC), but not the negative control (U1; Fig. 5g). Thus, our findings indicate that E2F1 acts as a transcription factor by binding to the Linc00337 promoter and regulating its expression in PDAC cells.

### Linc00337 binds to E2F1 in PDAC cells and acts as an E2F1 co-activator

**Fig. 6:**
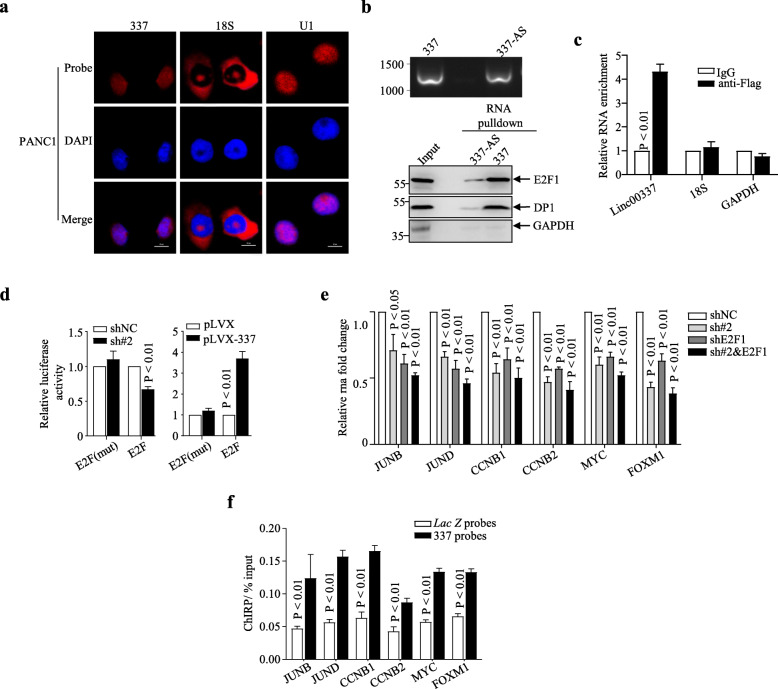
Linc00337 binds to E2F1 and acts as a E2F1 coactivitor. **a**, RNA FISH was carried out to determine the subcellular localization of Linc00337 in PANC1 cells. **b**, in vitro-transcribed biotin-labeled Linc00337 and its antisense RNA (top) and RNA pulldown assays showing that Linc00337 pulled down E2F1 and its partner DP1, but not GAPDH in PANC1 cells (bottom). **c**, Flag-E2F1 RIP assays suggesting that Linc00337 could be enriched by E2F1 in PANC1 cells. **d**, Stably-transfected PANC1 (left) and AsPC1 (right) cells were used to determine the transcriptional activity of E2F1. **e**, qRT-PCR analysis of E2F1 targets in indicated stably-transfected PANC1 cells. **f**, ChIRP-qRT-PCR detection of Linc00337 occupancy at the indicated E2F1 target loci. Data represent the mean ± SD of three independent experiments

We then investigated the subcellular localization of Linc00337 in PANC1 cells, finding that Linc00337 was mainly localized in the nucleus (Fig. 6a). Since E2F1 is a transcription factor that is localized in nucleus, we speculated that Linc00337 may bind to E2F1 and predicted their interaction probability using the RPISeq website (http://pridb.gdcb.iastate.edu/RPISeq/). Both the RF and SVM classifier probabilities (0.75 and 0.85, respectively; Fig. S4a) exceeded 0.5, suggesting that Linc00337 may bind to E2F1. Therefore, we performed biotin-labeled RNA pulldown assays which revealed that Linc00337 pulled down E2F1 and its partner DP1, but not the negative control GAPDH (Fig. 6b), while Flag-E2F1 RIP assays confirmed that Linc00337 binds to E2F1 in PANC1 cells (Fig. 6c and S4b).

We found that Linc00337 not only binds to E2F1 but also regulates its expression, while previous studies have shown that various lncRNAs modulate the binding efficiency of E2F1 to its target promoters or sponge endogenous miRNA expression to regulate E2F1 activity [28, 29]. Therefore, we investigated whether Linc00337 regulates the transcriptional activity of E2F1 using Luciferase reporter assays. As shown in Fig. 6d, E2F1 luciferase activity increased in AsPC1 cells following Linc00337 overexpression but decreased in PANC1 cells following Linc00337 knockdown. We further examined whether Linc00337 directly regulates the expression of E2F1 target genes. Consistently, the downstream targets of E2F1 were down-regulated following Linc00337, E2F1 and both of them knockdown in PANC1 cells (Fig. 6e).

To determine whether Linc00337 regulates the transcriptional activity of E2F1 by actively binding to chromatin and acting as a co-activator, we performed ChIRP assays. Probes complementary to Linc00337 were used to pull down endogenous Linc00337 from PANC1 cells and the promoter regions of known E2F1-binding sites were amplified and quantified by qRT-PCR (Fig. S4c). Consistent with the hypothesis that Linc00337 acts as an E2F1 co-activator, ChIRP analysis revealed that Linc00337 is recruited to a subset of E2F1 target promoters in PANC1 cells, suggesting overlapping chromatin occupancy between Linc00337 and E2F1 (Fig. 6f).

### Linc00337 via regulating E2F1 to exert its oncogenic roles in PDAC cells

**Fig. 7:**
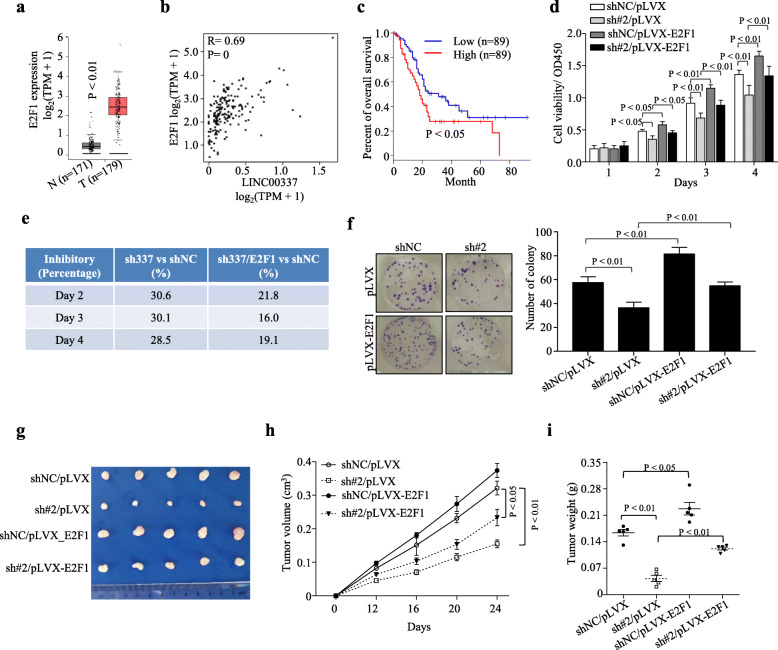
Linc00337 via regulating E2F1 to exert its oncogenic roles in PDAC cells. **a-c**, TCGA data revealing that E2F1 is highly expressed in PDAC tissues (**a**), correlates positively with Linc00337 (**b**), and predicts poor prognosis (**c**). **d-f**, E2F1 partly rescues cell proliferation repressed by Linc00337 knockdown in PANC1 cells as indicated by CCK8 cell viability assays (**d**), the inhibitory ratio (**e**), and colony formation assays (**f**). **g-i**: Xenograft subcutaneous tumor formation assays with indicated stably-transfected PANC1 cells showing tumor size (**g**), growth curve (**h**), weight (**i**). In (**d-f**), data represent the mean ± SD of three or four independent experiments

To investigate whether Linc00337 exerts its biological effects via E2F1, we retrieved information from the TCGA database. As expected, E2F1 levels were significantly elevated in PDAC tissues (Fig. 7a), correlated positively with Linc00337 expression (Fig. 7b), and predicted poor overall survival (Fig. 7c). Finally, both in vitro (Fig. 7d-f) and in vivo (Fig. 7g-i) rescue assays indicated that ectopic E2F1 expression attenuated the inhibition of proliferation caused by Linc00337 knockdown. Taken together, these results suggest that Linc00337 promotes PDAC cell proliferation and growth by binding to E2F1 and regulating its activity.

## Discussion

In this study, we reported Linc00337 not only overexpression in patients with PDAC but also closely corelated with clinicopathological parameters and further demonstrated that Linc00337 accelerates PDAC cell cycle transition and cell proliferation in vitro and tumor growth in vivo using loss- and gain-function assays. Moreover, increased Linc00337 expression correlated with poor clinical outcomes including OS and DFS, supporting our conclusion that Linc00337 exerts strong effects on PDAC cell proliferation and tumor growth.

LncRNAs play intricate roles via various different mechanisms, including the regulation of gene expression at the transcriptional and post-transcriptional levels [30–32], with increasing evidence suggesting that lncRNA deregulation is associated with tumorigenesis and progression. Here, we demonstrated that Linc00337 exerts oncogenic effects by regulating the expression of E2F1, a crucial oncogenic transcription factor that plays key roles in cell cycle progression and cell proliferation in various malignancies [9, 33]. Previous reports have identified several E2F1-associated lncRNAs, of which lncRNA-TINCR [34], lncRNA-KHPS1 [15], and Linc00668 [17] are direct targets of E2F1 and lncRNA-GASL1 [35] and lncRNA-H19 [36] are related to its activity. These E2F1-related lncRNAs have been reported to play extensive roles in different malignant tumors; however, the underlying mechanisms are poorly understood. In this study, we verified that E2F1 directly binds to the promoter of Linc00337 and triggers its expression. We noticed that Linc00337 regulates a lot of genes involved in the late cell cycle phase including KIFs and CDCs. While, PDAC cells stopping in G1 phase after LINC0037 silencing, we supposed that the G1/S arrest in PDAC cells after Linc00337 silencing mainly due to the inhibition of E2F1, as the roles of E2Fs are pivotal in G1/S transition in human cells.

Mechanistically, we demonstrated that Linc00337 acts as an E2F1 co-activator by interacting with E2F1 and regulates its transcriptional activity. Since this interaction facilitates the activation of the E2F1 transcriptional network, a Linc00337-dependent E2F1 response may act via a positive feedback mechanism wherein E2F1 induces Linc00337 expression which promotes E2F1 expression and enhances its activity. However, future studies should investigate how Linc00337 regulates E2F1 expression.

Linc00337 overexpression has been previously reported in lung [37] and gastric cancer tissues [19]. In gastric and lung cancer Linc00337 seems to decrease the transcription of target genes and bind to DNMT1 [21] or EZH2 [19]. Here we are showing a transcriptional upregulation of target genes by Linc00337. We showed that Linc00337 promotes PDAC progression by enhancing the transcriptional activity of E2F1. All of these findings illustrate the oncogenic roles of Linc00337 in different cancers and we can’t rule out the same mechanisms which observed in gastric and lung cancer could be found in PDAC. We noticed that recently Yang et al. reported that Linc00337 recruits E2F4 to up-regulate TPX2 and induces autophagy and chemoresistance to cisplatin in esophageal squamous cell carcinoma [22]. Their findings revealed that Linc00337 binds to E2F4, the other E2Fs family member [38], taken our results and their findings together, Linc00337 seems to be largely related to the E2Fs family members and it should be further studied in the further. Moreover, we also found that Linc00337 is also involved in PDAC cell chemoresistance and metastasis both in vitro and in vivo (data will be published in the future). Taken together, Linc00337 exerts its oncogenic effects in different aspects in PDAC and other cancers by multiple mechanisms which indicates Linc00337 might be a key regulator in tumorigenesis and cancer progression. Therefore, our findings add another level of complexity to Linc00337-mediated gene regulation in different cancers.

Previous studies have indicated that lncRNAs display potential advantages in cancer diagnosis and prognosis [39, 40]. In this study, we found that high Linc00337 levels were strongly associated with poor OS and DFS in PDAC patients; therefore, Linc00337 should be investigated further as an independent risk factor for PDAC patient survival.

## Conclusions

Taken together, our results demonstrate a reciprocal regulation mechanism between Linc00337 and E2F1 in PDAC progression. Clinically, Linc00337 displays a remarkable potential value for PDAC prognosis and have improved our understanding of its roles and clinical significance in PDAC and its molecular pathology to provide a potential therapeutic target for PDAC treatment.

## Supplementary information

**Additional file 1: Figure S1.** Box plots depicting the expression of 20 cancer-associated lncRNAs in PDAC and normal tissues. TCGA data was analyzed using the GEPIA website (http://gepia.cancer-pku.cn/index.html). **Figure S2.** Overall survival analysis of the expression of 20 cancer-associated lncRNAs in PDAC. TCGA data was analyzed using the GEPIA website (http://gepia.cancer-pku.cn/index.html). **Figure S3.** RNA decay curves depicting the degradation of mRNAs. 18S was used as a reference gene and no significant alterations were observed following Actinomycin D treatment. Data represent the mean ± SD of three independent experiments. **Figure S4.** a, RPISeq prediction parameters of Linc00337 and E2F1 binding. b, western blot analysis of Flag-E2F1 immunoprecipitation, DP1 as positive control and GAPDH as negative control. c, qRT-PCR analysis of Linc00337 and GAPDH enrichment in PANC1 cells by *Lac Z* and *337.*

## Data Availability

The data used and analyzed during the current study are available from the corresponding author on reasonable request.

## Abbreviations

LncRNA: Long noncoding RNA
PDAC: Pancreatic adenocarcinoma
RIP: RNA-immunoprecipitation
FISH: Fluorescence in situ hybridization
TCGA: The Cancer Genome Atlas
qRT-PCR: quantitative real-time PCR
ChIP: Chromatin immunoprecipitation
ChIRP: Chromatin isolation by RNA purification
OS: Overall survival
DFS: Disease-free survival
CPAT: Coding potential assessing tool
