# Supplementary material for: Long noncoding RNA Linc00337 functions as an E2F1 co-activator and promotes cell proliferation in pancreatic ductal adenocarcinoma
Source: J Exp Clin Cancer Res. 2020 Oct 14;39:216. doi: 10.1186/s13046-020-01725-5 (PMC7557102; doi:10.1186/s13046-020-01725-5)
Supplement: Supplementary file 1 — Additional file 1: Figure S1. Box plots depicting the expression of 20 cancer-associated lncRNAs in PDAC and normal tissues. TCGA data was analyzed using the GEPIA website (http://gepia.cancer-pku.cn/index.html). Figure S2. Overall survival analysis of the expression of 20 cancer-associated lncRNAs in PDAC. TCGA data was analyzed using the GEPIA website (http://gepia.cancer-pku.cn/index.html). Figure S3. RNA decay curves depicting the degradation of mRNAs. 18S was used as a reference gene and no significant alterations were observed following Actinomycin D treatment. Data represent the mean ± SD of three independent experiments. Figure S4. a, RPISeq prediction parameters of Linc00337 and E2F1 binding. b, western blot analysis of Flag-E2F1 immunoprecipitation, DP1 as positive control and GAPDH as negative control. c, qRT-PCR analysis of Linc00337 and GAPDH enrichment in PANC1 cells by Lac Z and 337. [file 13046_2020_1725_MOESM1_ESM.pdf]

Supplementary figures

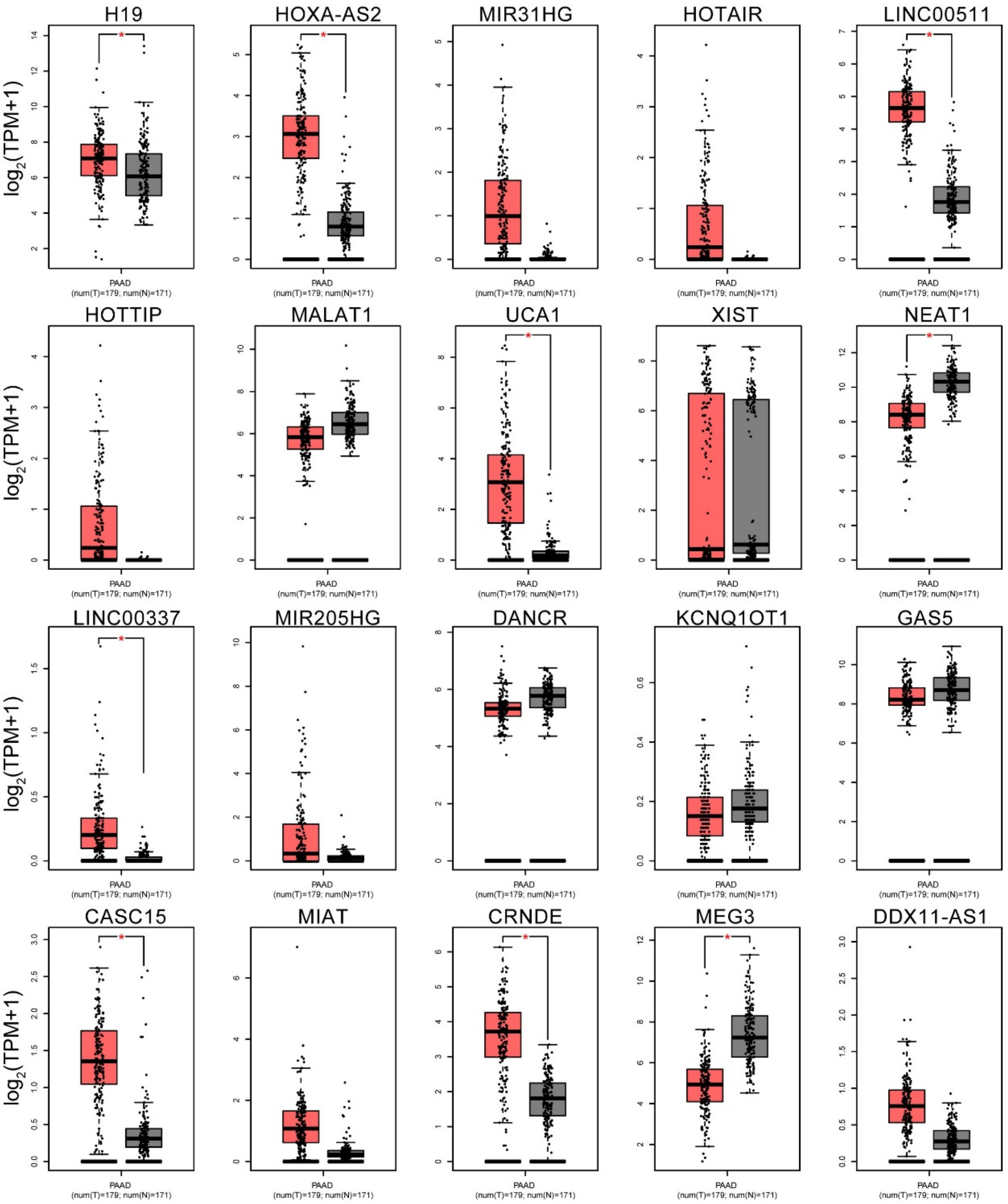

Figure S1. Related to Figure 1

Box plots depicting the expression of 20 cancer-associated lncRNAs in PDAC and normal tissues. TCGA data was analyzed using the GEPIA website (http://gepia.cancer-pku.cn/index.html).

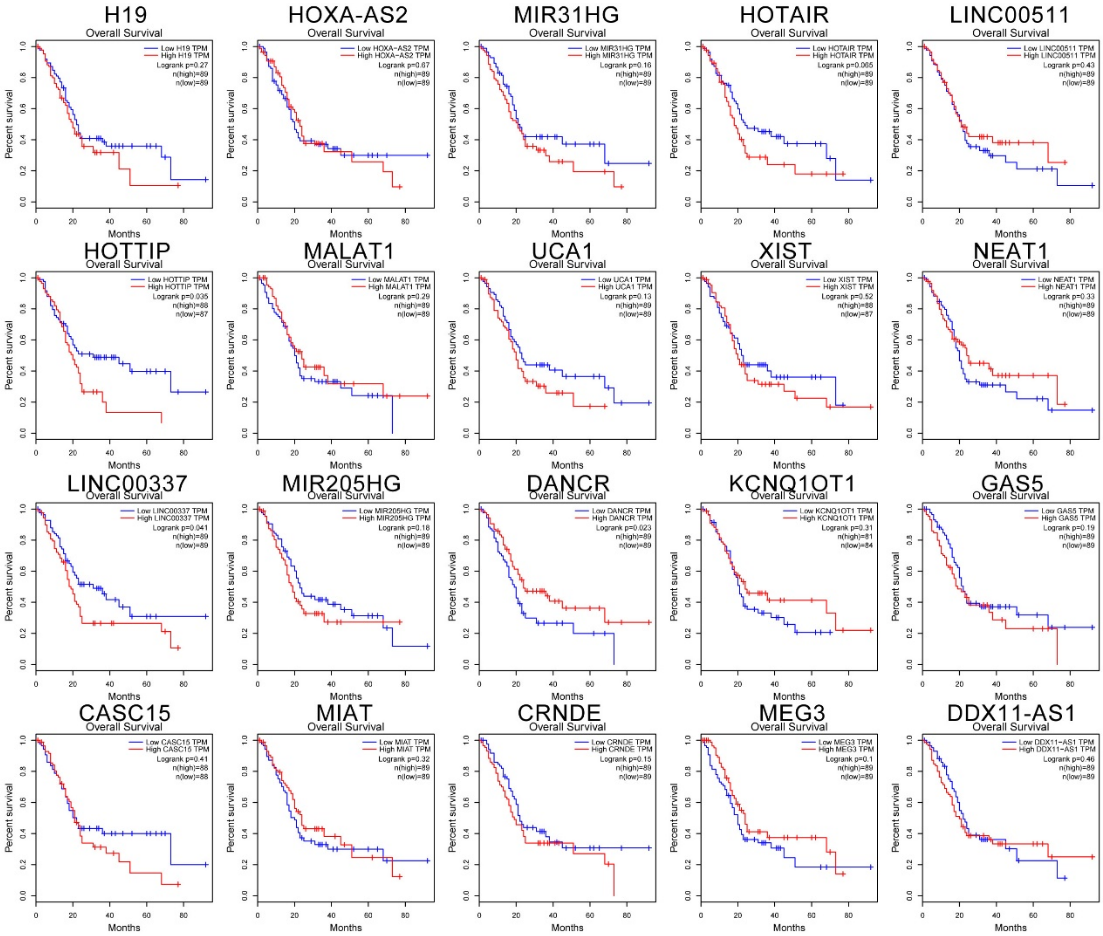

Figure S2. Related to Figure 1

Overall survival analysis of the expression of 20 cancer-associated lncRNAs in PDAC. TCGA data was analyzed using the GEPIA website (http://gepia.cancer-pku.cn/index.html).

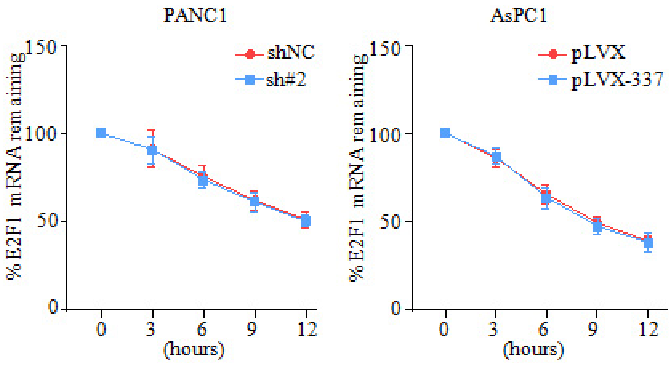

Figure S3. Related to Figure 4

RNA decay curves depicting the degradation of mRNAs. 18S was used as a reference gene and no significant alterations were observed following Actinomycin D treatment.

Data represent the mean ± SD of three independent experiments.

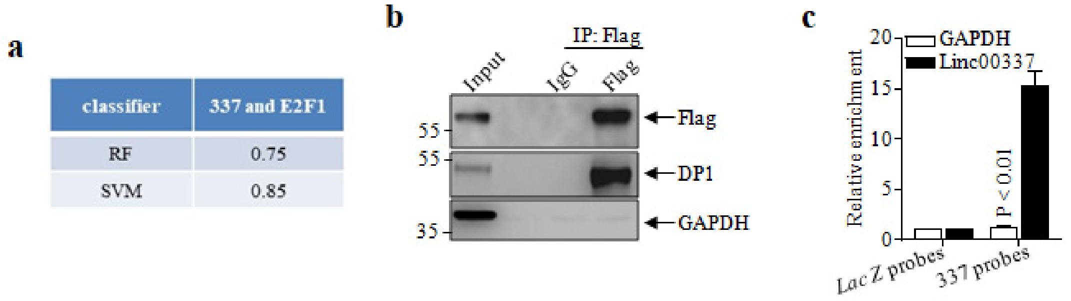

Figure S4. Related to Figure 5

a, RPISeq prediction parameters of Linc00337 and E2F1 binding.

b, western blot analysis of Flag-E2F1 immunoprecipitation, DP1 as positive control and GAPDH as negative control.

c, qRT-PCR analysis of Linc00337 and GAPDH enrichment in PANC1 cells by *Lac Z* and *337*.
